# Digestibility, ruminal enzyme activity, fermentation characteristics, and selected blood biochemical parameters in calves supplemented with humic acid

**DOI:** 10.3389/fvets.2025.1704848

**Published:** 2026-01-05

**Authors:** Waleed K. Abouamra, Abdelslam M. A. Amhabj, Hamdan M. Tawfik, Ahmed A. Ayad, Mohsen M. Farghaly, Hayam M. A. Monzaly, Mohsen A. Khormi, Ramadan Taha, Ahmed Ezzat Ahmed, Amira K. Hajri, Marzough Aziz Albalawi, Montaser Elsayed Ali

**Affiliations:** 1 Department of Animal Production, Faculty of Agriculture, Al-Azhar University, Assiut, Egypt; 2 Department of Animal Production, Faculty of Agriculture, Sirte University, Sirte, Libya; 3 Department of Animal Production, Faculty of Agriculture, Assiut University, Assiut, Egypt; 4 Sheep and Goat Research Department, Animal Production Research Institute, Agriculture Research Centre, Giza, Egypt; 5 Department of Biology, College of Science, Jazan University, Jazan, Saudi Arabia; 6 Department of Biology, College of Science, King Khalid University, Abha, Saudi Arabia; 7 Prince Sultan Bin Abdelaziz for Environmental Research and Natural Resources Sustainability Center, King Khalid University, Abha, Saudi Arabia; 8 Department of Chemistry, Alwajh College, University of Tabuk, Tabuk, Saudi Arabia

**Keywords:** digestibility, feed intake, growth performance, humic acid, ruminal fermentation

## Abstract

This study aimed to evaluate the effect of humic acid (HA) on growth performance, nutrient digestibility, ruminal fermentation, ruminal enzyme activity, and certain blood biochemical parameters. A total of 45 calves (11 ± 0.25 months of age; 280 ± 5.55 kg BW) were divided equally into three groups. The control group received a basal diet composed of a concentrate feed mixture (CFM) with roughage. The treatment groups received the same basal diet supplemented with 1 and 2% humic acid in the CFM, respectively. The study revealed that the treatment groups had significantly lower total dry matter (DM) intake (*p* = 0.029). The treatment groups showed higher (*p* = 0.013) ether extract (EE) digestibility and digestible crude protein (DCP) (*p* = 0.001) than the CFM group. However, ammonia nitrogen (NH_3_-N, mg/100 mL) was significantly lower (*p* = 0.012) in the 1% HA and 2% HA groups compared to the CFM group. The 2% HA group had significantly higher (*p* = 0.001) proportions of acetate and propionate compared to the 1% HA and CFM groups. The treatment groups had significantly higher (*p* = 0.001) *α*-amylase, lipase, urease, and protease activities compared to the CFM group. In addition, the concentration of total soluble protein in the rumen liquid was higher (*p* = 0.006) in the 1% HA group compared to the CFM group. The 2% HA group had higher (*p* = 0.049) glucose concentrations than the CFM group. In conclusion, humic acid supplementation at 1% or 2% may improve nutrient digestibility and ruminal fermentation efficiency, enhance enzyme activity, and support normal blood profiles in calves.

## 1 Introduction

In the context of global nutritional challenges, Egypt’s livestock sector represents a vital part of world animal production. Its contribution extends beyond national food security and economic stability (1), providing essential nutrients and economic resilience for millions of people (2). The sector’s production of food and raw materials, coupled with its function as a financial safety net through the sale of live animals and products (3, 4), highlights its importance in the broader effort to meet global dietary needs. Despite its importance, Egypt has experienced a notable decline in its cattle population, from 4.5 million in 2018 to 2.8 million in 2021. This decline is largely due to disease outbreaks and a combination of interacting factors, including host susceptibility, pathogen characteristics, environmental stressors, and management practices (5).

Most cattle (61%) are raised in small-scale herds, which increases their vulnerability to health and nutritional challenges (6). Rising nutritional ingredient costs, land scarcity, and climate change have intensified interest in feed additives that can enhance calf performance and improve feed utilization efficiency (7). Organic acids (OAs) are used to enhance the effectiveness of beneficial ruminal bacteria during fermentation and are “generally recognized as safe” and authorized for animal feeding by the European Union (8). Hence, OAs influence rumen digestion and protein breakdown, leading to improved feed intake, growth performance, and feed conversion ratio (FCR) (9).

Humic compounds, including humic acid (HA), are gaining attention for their antimicrobial and anti-inflammatory properties, with the potential to benefit animal health by reducing inflammation and promoting overall wellbeing (10, 11). In the context of animal nutrition, HA is a complex organic molecule that primarily functions in the rumen as a prebiotic and antimicrobial agent (12). It enhances the activity of fiber-digesting bacteria and reduces methane production, optimizing fermentation efficiency and contributing to improved livestock productivity and sustainability (13). Chemically, HA is defined as a complex mixture of aliphatic and aromatic compounds containing various functional groups (14). Its structure is primarily composed of phenolic, carboxylic acid, enolic, quinone, and other functional groups, although it may also contain sugars and peptides and can be recovered from several sources (15). In monogastric animals, HA has shown promising results, improving growth, immune response, and nutrient digestibility (16). However, inconsistent results have been reported in ruminant studies (17).

Humic acid (HA) has gained considerable attention for its potential to reduce nitrogen losses in the rumen by binding nitrogenous compounds such as ammonia (15, 16). Therefore, it enhances nitrogen retention, promotes microbial protein synthesis, and improves ruminal nitrogen utilization efficiency (17). Several studies have demonstrated that HA improves gut health, increases nutrient utilization, and positively influences rumen microbial activity by stabilizing pH and supporting fermentation efficiency (18). However, other research has found no significant effects on volatile fatty acid (VFA) production, ruminal pH, or methane emission (19). Furthermore, HA may affect metabolic function and overall animal health by changing critical blood metabolites, thereby contributing to improved growth and performance outcomes (20).

We hypothesized that dietary supplementation with 1% or 2% HA would improve overall growth performance and feed efficiency in calves by enhancing nutrient digestibility, optimizing ruminal fermentation patterns, and stimulating ruminal enzyme activity and certain blood biochemical parameters. Therefore, this study aimed to evaluate the effect of dietary HA supplementation on growth performance, feed intake, feed conversion ratio, nutrient digestibility, ruminal fermentation parameters, ruminal enzyme activity, and certain blood biochemical parameters in calves.

## 2 Materials and methods

### 2.1 Location and ethical approval

The study was conducted from January 2024 to March 2025 on a private farm in the Western Desert of Egypt, located 320 kilometres northeast of Cairo, in the city of Al-Qusiya, Assiut Governorate, away from the Nile River (geographically between latitude 27°28′32.0″N and longitude 30°33′31.6″E). All animal owners provided informed consent before the animals were included in the study. The study protocol was reviewed and approved by the Research Ethics Committee of the Faculty of Agriculture, Assiut University (Ref. No: 03–2025-0039).

### 2.2 Animal design and management

A total of 45 crossbred male calves (Friesian × local breed) were included in this study. All animals were confirmed to be clinically healthy based on a veterinary examination prior to the experiment. The calves were 11 ± 0.25 months old, with a mean body weight of 280 ± 5.55 kg (mean ± SD). In the same barn, the animals were randomly assigned to three dietary treatments (15 calves per group). The control group received a basal diet composed of a concentrate feed mixture (CFM) with alfalfa hay, corn silage, and wheat straw as roughage. The treatment groups received the same basal diet, but the CFM was supplemented with 1% or 2% humic acid (1% HA and 2% HA groups, respectively). The HA was sourced from the Al-Gomhoria Chemicals Company (Cairo, Egypt). During the manufacturing process of the CFM, HA was mechanically blended with the other ingredients and pelleted to ensure homogeneous distribution in the final diet.

#### 2.2.1 Feeding trial

The feeding trial lasted 134 days, which included a preliminary adaptation period of 14 days followed by a 120-day experimental phase. Body weights were recorded at the beginning of the experiment and at 30-day intervals before the morning feeding. All animals received 70% of their nutritional requirements from the concentrate mixture, with the remaining 30% provided by roughage components (alfalfa hay, corn silage, and wheat straw). Each group was housed in a semi-open, well-ventilated barn (10 × 5 m) equipped with individual feeding stalls during feeding periods to accurately record individual feed intake and provided with automatic drinkers.

The quantity of concentrate offered was adjusted monthly according to changes in body weight, following the National Research Council (NRC, 2001) nutritional guidelines (21) and beef cattle recommendations. Feed remainders were collected individually and weighed daily to determine daily feed intake. The animals were weighed at the start and end of the experiment, as well as monthly throughout the study. The mean average daily gain (ADG) was calculated as the difference between the final and initial body weights divided by the total number of feeding days.

The feed conversion ratio was calculated and expressed in terms of kg of dry matter (DM), total digestible nutrients (TDN), and digestible crude protein (DCP) per unit of body weight gain. Vitamin and mineral blocks, as well as fresh water, were provided ad libitum. The concentrate mixture was offered separately twice daily at 8:00 a.m. and 8:00 p.m., while roughage was provided *ad libitum* throughout the day. Acid-insoluble ash (AIA) was determined according to the method described by Van Keulen and Young (22). The ingredients and chemical composition of the concentrate mixture, as well as the inclusion of corn silage, alfalfa hay, and wheat straw in the experimental diets, are presented in Table 1.

**Table 1:** Ingredients and chemical composition of the concentrate mixture, corn silage, alfalfa hay, and wheat straw used in the experimental diets (%).

Items (%)	Experimental concentrate mixture diets			Corn silage	Alfalfa hay	Wheat straw
	CFM	1% HA	2% HA			
The ingredients						
Maize grain	45	44	44	–	–	–
Soybean meal	21	21	21	–	–	–
Gloved	15	15	14.5	–	–	–
Wheat bran	12.5	12.5	12	–	–	–
Gluten	2.5	2.5	2.5	–	–	–
Limestone	1.7	1.7	1.7	–	–	–
Salt	1.2	1.2	1.2	–	–	–
Humic acid	–	1	2	–	–	–
Trace mineral and vitamin premix^*^	0.3	0.3	0.3	–	–	–
Buffer	0.4	0.4	0.4	–	–	–
Yeast	0.4	0.4	0.4	–	–	–
Chemical composition						
Dry matter (DM)	89.7	90	90.1	32.5	89.34	86.84
Organic matter (OM)	92.70	92.2	92.5	90.66	90.82	83.90
Crude protein (CP)	15.34	15.7	15.6	8.43	18.78	3.68
Crude fiber (CF)	14.8	13.8	14.1	22.8	21.45	35.23
Ether extract (EE)	2.1	2.3	2.2	2.67	4.03	1.56
Nitrogen-free extract (NFE)	60.46	60.4	60.6	56.76	46.56	41.54
Ash	7.30	7.80	7.50	9.34	9.18	13.34
Acid insoluble ash (AIA)	1.70	1.80	1.60	1.78	1.32	1.07

CFM, control group. 1% HA = calves received 1% humic acids, 1% HA = calves received 2% humic acids. *The premix contained (per kg) 20,000,000 IU of vitamin A, 200,000 IU of vitamin D3, 10,000 mg of vitamin E, 10,000 mg of Fe, 2,500 mg of Cu, 20,000 mg of Mn, 100 mg of Mo, 100 mg of Co, 800 mg of I, 20,000 mg of Zn, and 100 mg of Se.

### 2.3 Digestibility trial

Over the course of 7 days, fecal samples were collected from 10 calves in the CFM, 1% HA, and 2% HA groups at the end of the feeding trial. Twice a day, 200 g of fresh feces were obtained directly from the animals’ rectums at the same time for all groups using the (individual) fecal grab technique for seven consecutive days, and they were promptly refrigerated (49). Furthermore, nutrient digestibility coefficients were calculated using acid-insoluble ash (AIA) as an internal marker (22). At the end of the collection period, the fecal samples from each animal were pooled, combined, homogenized, dried for 24 h at 60 °C, and ground to pass through a 1 mm sieve for subsequent chemical analysis. The Association of Official Analytical Chemists (50) standard operating protocols were used to assess the chemical composition of both feed and fecal samples. Nutrient digestibility was calculated using the following equation:


$$\begin{array}{l} \text{Digestibility}\;(\%)=100-\frac{\mathrm{AIA}\;\text{concentration in diets}\;}{\mathrm{AIA}\;\text{concentration in feces}}\times \\ \frac{\text{Anutrient concentration in feces}\;\;}{\text{nutrient concentration in diets}} \end{array}$$

### 2.4 Rumen liquid parameters

Using a stomach tube, samples of rumen contents were obtained from 10 calves in each group at the end of the digestibility trial. Samples were collected once every 4 h after feeding. The samples of rumen fluid were divided into two parts: (i) the first part was filtered through a single layer of cheesecloth to determine the total protozoa count (×106/mL) (23) and (ii) the second part was filtered through four layers of cheesecloth and used to measure pH with a digital pH meter (Beckman, model 45, USA).

Ammonia nitrogen (NH_3_-N, mg/100 mL) in the rumen fluid was analyzed using an atomic absorption spectrophotometer (24). Before being stored for analysis, the strained rumen liquid samples were acidified with 0.1 N hydrochloric acid and 2–3 drops of formalin or formaldehyde to inhibit microbial activity.Then, the samples were kept frozen at −20 °C for the determination of total volatile fatty acid (VFAs) concentration (mmol/100 mL).

Gas chromatography (GC) was used to measure the molar proportions of VFAs, including acetate, propionate, n-butyrate, iso-butyrate, n-valerate, iso-valerate, and acetate/propionate (25). The analysis was performed on a Carlo Erba 5,000 model (Milan, Italy) equipped with a DB-FFAP column (or equivalent; 15 m, 0.53 mm ID, 1 μm). Hydrogen served as the carrier gas (head pressure: 15 kPa). The temperature program started at 50 °C for 1 min, followed by a 30 °C/min ramp to 220 °C, with a final hold time of 5 min. Injector and detector temperatures were maintained at 250 °C. The samples were analyzed in triplicate; the first result was discarded, and the results were calculated as the mean of the replicates.

### 2.5 Ruminal enzyme activities

A subsample of 5 mL from the whole samples of rumen liquid, collected 4 h after the morning feeding from 10 calves in each treatment, was preserved by adding a few drops of saturated mercuric chloride solution to stop microbial activity and stored at −20 °C for the determination of ruminal enzyme activities. Enzyme activities in rumen fluid were measured spectrophotometrically (Unico, USA).

Ruminal enzyme activities were determined using colorimetric methods. *α*-Amylase activity was measured using the 3,5-dinitrosalicylic acid (DNS) method (26); 0.1 mL of rumen fluid was incubated with 0.9 mL of 1% soluble starch in 50 mM phosphate buffer (pH 6.8) at 37 °C for 30 min. The reaction was stopped by adding 1.0 mL of DNS reagent, followed by boiling for 5 min. After cooling, absorbance was measured at 540 nm (27). One unit of *α*-amylase activity was defined as μg glucose/minute. The standard curve was prepared using a serial dilution of glucose.

The essential reagents for the determination of cellulase activity were a glucose stock solution (10 mg/mL), 0.05 M citrate buffer (pH 4.8), dinitrosalicylic acid (DNS), and a 1% carboxymethyl cellulose solution (1 g of cellulose dissolved in 0.05 M citrate buffer). One milliliter of the tested sample (extracellular enzymes from rumen microbiota) was added to a tube containing 1 mL of 1% carboxymethyl cellulose solution, then the reaction mixture was incubated at 37 °C for 1 h under unstirred conditions. The reaction was terminated by adding 3 mL of DNS, and the tubes were placed in a boiling water bath for 5 min (28). A blank was taken where the enzyme was deactivated by adding 3 mL DNS before the commencement of incubation. The reaction mixture was cooled to 30 °C, and 5 mL of distilled water was added to each tube to bring the final volume to 10 mL. The mixture was centrifuged at 5,000 rpm for 5 min, and the intensity of color was measured at 540 nm using a spectrophotometer (27). One unit of cellulase activity was defined as the amount of enzyme that produces 1 μg of reducing sugar (expressed as glucose) per minute under the standard assay conditions. Activity is reported as units per milliliter of the tested sample filtrate. Activity is reported as units per milliliter of the tested sample filtrate. The standard curve was prepared using a serial dilution of glucose from a glucose stock (10 mg/mL).

Lipase activity was assayed using p-nitrophenyl palmitate (p-NPP) as described previously (29). First, solution A (40 mg of p-NPP dissolved in 12 mL of isopropanol) and solution B (0.4 mL of Triton X-100 dissolved in 90 mL of distilled water) were prepared. Then, the substrate solution was prepared by adding 1 mL of solution A dropwise to 19 mL of solution B with constant stirring to form a stable emulsion, which remained stable for 2 h. The reaction mixture included 1 mL of the substrate solution, 0.5 mL of buffer (Tris buffer, pH 7, 100 mM), and 1 mL of the supernatant (crude enzyme), with distilled water added to bring the final volume to 3 mL. The mixture was incubated for 45 min at 30 °C. Enzyme activity was stopped by adding 0.2 mL of isopropanol. Absorbance was measured spectrophotometrically at 410 nm against a free blank that contained distilled water instead of the tested sample (crude enzyme).

The standard curve was prepared using para–nitrophenol. One unit of enzyme activity was defined as μg p-NPP / minute using 1 mL of the tested sample under standard assay conditions (30). Protease activity was estimated using a method described previously (31). The reaction mixture containing 5 mL of casein and 1 mL of crude enzyme was mixed and incubated at 30 °C for 10 min. The reaction was stopped by adding 5 mL of trichloroacetic acid, followed by the addition of 5 mL of Na_2_CO_3_ and 1 mL of the Folin–Ciocalteu reagent. Absorbance was measured at 660 nm, and the amount of liberated amino acids was calculated using the standard curve of tyrosine. Urease activity was determined using the phenol-hypochlorite method described by a previous study (32).

The reaction mixture, containing 1 mL of the tested sample, 1 mL of 100 mM phosphate buffer (pH 6.7), and 1 mL of 50 mM urea, was incubated at 37 °C for 30 min. The reaction was stopped by adding 1 mL of a 5.0% (w/v) phenol-nitroprusside solution. Then, 500 μL of a 0.2% alkaline hypochlorite solution was added, and the mixture was shaken for 10 min at room temperature. Ammonia concentrations generated from the urease reaction were determined using a spectrophotometer at 625 nm (33). One unit of urease activity was defined as the amount of enzyme that produces 1 μg of ammonium (NH_4_+) per minute under the standard assay conditions (34). Activity is expressed as units per milliliter of rumen filtrate. The standard curve was prepared using a serial dilution of an NH_4_CL stock solution (1,000 mg/mL). The concentration of extracellular protein in the crude enzyme was measured as described previously by Lowry et al. (35). The protein concentration in the tested samples was calculated as mg/mL. The standard curve was prepared using bovine serum albumin.

### 2.6 Blood biochemical analysis

On the final day of the feeding trial (day 120), after the morning feeding, 45 blood samples (15 animals × 3 groups) were collected from the jugular vein of each animal. After centrifugation at 3,000 × g for 20 min, serum samples were separated and stored at −20 °C until further analysis. Blood glucose (mg/dL) concentrations were determined using sodium fluoride-coated collection tubes. Biochemical parameters, including glucose (mg/dL), triglyceride (mg/dL), cholesterol (mg/dL), and urea (mg/dL), were measured using commercial assay kits (Diamond Chemical Company, Germany) (36). Total protein (g/dL), albumin (g/dL), and globulin (g/dL) concentrations were measured using specific kits (Spinreact Company, Spain) (37). Furthermore, creatinine (mg/dL) and aspartate transaminase (AST) activity were measured using assay kits (Spectrum Chemical Company, Egypt) (38).

### 2.7 Statistical analysis

Data were analyzed using the general linear model (GLM) procedure of SAS (2001). For variables measured repeatedly (e.g., body weight and feed intake), a mixed model (PROC MIXED) was applied, including the fixed effects of treatment, time, and their interaction, with animal included as a random effect. Normality and homogeneity of variance were assessed using the Shapiro–Wilk test and Levene’s test. Initial body weight was included as a covariate in growth-related analyses when it improved model fit. The following model was used: Yijkl = *μ* + Ti + Dj + (T × D)ij + *β*(Initial BW) + Ak(i) + eijkl, where *μ* is the overall mean, Ti is the effect of treatment, Dj is the effect of day, (T × D)ij is the interaction, *β* is the regression coefficient of the covariate, Ak(i) is the random effect of animal, and eijkl is the residual error. Variables measured once (e.g., nutrient digestibility, ruminal fermentation, enzyme activity, and blood biochemistry) were analyzed using one-way ANOVA (PROC GLM), and means were compared using Duncan’s multiple range test (39). When the *F*-test was significant at a *p*-value of < 0.05, means were compared using Duncan’s multiple range test. The following model was used: Yij = *μ* + Ti + eij, where Yij is the experimental observation, μ is the general mean, Ti is the effect of treatment, i is the control, T1, and T2, and eij is the error related to the individual observation. The data were presented as means ± SEM. Probability values (*F*-values) of less than 0.05 (*p* < 0.05) were considered significant. Units were standardized (e.g., kg head^−1^ day^−1^), and all equipment and reagent manufacturers were reported along with their city and country.

## 3 Results

### 3.1 Growth performance, feed intake, and feed conversion

The results shown in Table 2 indicate that no differences (*p* > 0.05) were observed in initial weight (IW), final weight (FW), body weight gain (BWG), or average daily gain (ADG) between the calves supplemented with 1% or 2% humic acid and those in the CFM group. Furthermore, dry matter intake (DMI) of concentrates and total DMI were lower (*p* = 0.001) in the 1% HA and 2% HA groups compared to the CFM group, while DMI of hay was higher (*p* < 0.01) in the dietary treatment groups than in the CFM group. However, total intake in terms of total digestible nutrients (TDN) was higher (*p* = 0.022) in the 1% HA group compared to the 2% HA group. Moreover, digestible crude protein (DCP) intake was increased (*p* = 0.011) in the humic acid-supplemented groups compared to the control group. The feed conversion ratio (FCR) based on dry matter (DM), TDN, and DCP was improved (*p* = 0.001) in the humic acid treatment groups compared to the control group.

**Table 2:** Growth performance, feed intake, and feed conversion ratio of the calves supplemented with 1 and 2% humic acid compared to the CFM group.

Items	Treatment			*p*-value			
	CFM	1% HA	2% HA	SEM	Treat.	Time	Treat.^*^ time
Growth performance (Kg)							
Initial weight (IW)	280.55	280.22	279.22	10.05	0.999	0.734	0.878
Final weight (FW)	423.89	444.22	438.00	13.91	0.635	0.278	0.586
Body weight gain (BWG)	143.33	164.00	158.78	7.31	0.110	0.438	0.461
Average daily gain (ADG)	1.19	1.37	1.32	0.06	0.110	0.438	0.461
Feed intake (kg head^−1^ day^−1^)							
Dry matter intake (DMI) of concentrate	4.60^a^	3.11^b^	3.06^b^	0.05	0.001	0.621	0.341
Dry matter intake (DMI) of wheat straw	0.98	0.92	0.92	0.04	0.585	0.469	0.452
Dry matter intake (DMI) of hay	1.60^b^	2.30^a^	2.30^a^	0.06	0.001	0.243	0.561
Dry matter intake (DMI) of corn silage	2.17	2.28	2.24	0.04	0.226	0.854	0.785
Total dry matter intake (DMI)	9.35^a^	8.61^b^	8.53^b^	0.18	0.029	0.053	0.321
Total digestible nutrient (TDN) intake	6.48^a,b^	6.60^a^	6.41^b^	0.24	0.022	0.245	0.514
Digestible crude protein (DCP) intake	0.96^b^	1.05^a^	1.01^a^	0.10	0.011	0.011	0.721
Feed conversion ratio (FCR; kg/kg gain)							
Dry matter (DM)	7.86^a^	6.28^b^	6.46^b^	0.21	0.001	0.035	0.003
Total digestible nutrients (TDN)	5.45^a^	4.82^b^	4.86^b^	0.31	0.002	0.041	0.082
Digestible crude protein (DCP)	0.81^a^	0.77^b^	0.77^b^	0.02	0.025	0.021	0.062

^a,b^Means of the same row in each item with different superscripts are significantly different (*p*<0.05). CFM, control group. 1% HA=calve received 1% humic acids, 1% HA=calves received 2% humic acids.*Indicates the statistical interaction between treatments and times.

Nevertheless, growth performance and feeding intake were not influenced by time throughout the experiment or by the treatment × time interaction. However, the feed conversion ratio differed significantly (*p* = 0.035) over the experimental period. In addition, the treatment × time interaction had a more pronounced effect on the FCR in terms of DMI/kg gain.

### 3.2 Nutrient digestibility and nutritive value

The data presented in Table 3 show that there was no significant difference (*p* > 0.05) in nutrient digestibility and nutritive value, including dry matter (DM), organic matter (OM), crude protein (CP), crude fiber (CF), nitrogen-free extract (NFE), and total digestible nutrients (TDN), among the groups, although all metrics were improved in the dietary treatment groups compared to the CFM group. The HA-supplemented groups had significantly higher (*p* = 0.001) ether extract (EE) (*p* = 0.013) and digestible crude protein (DCP) compared to the CFM group.

**Table 3:** Nutrient digestibility and nutritive value of the calves supplemented with 1 and 2% humic acid compared to the CFM group.

Items (%)	Treatment			SEM	*p*-value
	CFM	1% HA	2% HA		
Dry matter (DM)	92.72	94.44	95.27	2.45	0.588
Organic matter (OM)	69.63	71.77	74.09	3.07	0.325
Crude protein (CP)	67.01	75.74	72.17	1.72	0.203
Crude fiber (CF)	57 0.83	53.26	51.47	1.96	0.351
Either extract (EE)	64.67^b^	75.91^a^	72.33^a^	3.39	0.013
Nitrogen-free extract (NFE)	81.46	83.42	84.70	2.58	0.605
Total digestible nutrients (TDN)	69.28	76.69	75.20	3.08	0.282
Digestible crude protein (DCP)	10.25^b^	12.16^a^	11.85^a^	0.01	0.001

^a,b^Means within the same row for each item with different superscripts are significantly different (*p* < 0.05). CFM, control group. 1% HA = calves received 1% humic acid, 2% HA = calves received 2% humic acid.

### 3.3 Ruminal fermentation parameters

The results presented in Table 4 show that there was no significant difference (*p* > 0.05) in ruminal pH values among the groups.

**Table 4:** Rumen liquid parameters of the calves supplemented with 1 and 2% humic acid compared to the CFM group.

Items	Treatment			SEM	*p*-value
	CFM	1% HA	2% HA		
pH	6.21	6.11	6.17	0.12	0.142
Ammonia nitrogen (NH_3_-N, mg/100 mL)	24.42^a^	16.34^b^	14.65^b^	0.68	0.012
Total VFA concentration (mmol/100 mL)	11.23	10.72	11.05	0.19	0.153
Relative proportion %					
Acetate	60.74^b^	61.64^a^	61.84^a^	0.35	0.001
Propionate	17.11^b^	17.18^b^	18.16^a^	0.24	0.001
n-Butyrate	15.57^a^	14.71^b^	14.46^b^	0.33	0.001
Iso-Butyrate	2.75^a^	2.56^a^	1.83^b^	0.24	0.001
n-Valerate	1.75	1.87	1.60	0.03	0.064
Iso-Valerate	2.08	2.03	2.11	0.18	0.162
Acetate/Propionate	3.55	3.59	3.41	0.07	0.138
Total protozoa count (×106/mL)	4.14	3.89	3.97	0.53	0.151

^a,b^Means within the same row for each item with different superscripts are significantly different (*p* < 0.05). CFM, control group. 1% HA = calves received 1% humic acid, 2% HA = calves received 2% humic acid.

Furthermore, ammonia nitrogen (NH_3_-N, mg/100 mL) was lower (*p* = 0.012) in the HA-supplemented groups compared to the CFM group. The molar proportions of VFAs, including acetate, propionate, n-butyrate, and iso-butyrate, varied significantly (*p* = 0.001) among the groups, although there was no significant difference (*p* > 0.05) in the concentration of total VFAs (mmol/100 mL). The 2% HA group had significantly higher proportions of acetate and propionate (*p* = 0.001) and lower proportions of n-butyrate and iso-butyrate (*p* = 0.001) compared to the 1% HA and CFM groups. In addition, the 1% HA group had higher proportions of acetate (*p* = 0.001) and lower proportions of n-butyrate (*p* = 0.001) compared to the CFM group. In contrast, there was no difference (*p* > 0.05) in the molar proportions of VFAs, including n-valerate, iso-valerate, and acetate/propionate, and the total protozoa count (×106/mL), among the groups.

### 3.4 Ruminal enzyme activity

*α*-Amylase activity (μg glucose/min/mL), lipase activity (μg p-nitrophenol/min/mL), urease activity (μg NH_3_/min/mL), and protease activity (μmol tyrosine/min/mL) were increased (*p* = 0.001) in the HA-supplemented groups compared to the CFM group (Table 5).

**Table 5:** Ruminal enzyme activities of the calves supplemented with 1 and 2% humic acid compared to the CFM group.

Items	Treatments			SEM	*p*-value
	CFM	1% HA	2% HA		
*α*-Amylase activity (μg glucose/min/mL)	4.73^b^	5.71^a^	5.88^a^	0.08	0.001
Cellulase activity (μg glucose/min/mL)	3.03^b^	4.46^a^	4.12^a,b^	0.01	0.021
Lipase activity (μg p-nitrophenol/min/mL)	5.34^b^	7.29^a^	6.22^a^	0.22	0.031
Urease activity (μg NH_3_/min/mL)	34.47^c^	50.20^a^	44.04^b^	0.71	0.001
Protease activity (μmol of tyrosine/min/mL)	3.84^b^	4.78^a^	4.94^a^	0.21	0.020

^a,b^Means within the same row for each item with different superscripts are significantly different (*p* < 0.05). CFM, control group. 1% HA = calves received 1% humic acid, 2% HA = calves received 2% humic acid.

Furthermore, cellulase activity (μg glucose/min/mL) was higher (*p* = 0.001) in the 1% HA group compared to the CFM group. No significant difference (*p* > 0.05) was observed in cellulase activity (μg glucose/min/mL) and urease activity (μg NH_3_/min/mL) between the 2% HA and the CFM groups. For rumen protein (mg/mL), there was a higher (*p* = 0.006) concentration in the 1% HA group than in the CFM group. In addition, there was no significant difference (*p* > 0.05) between the 2% HA and the CFM groups (Table 5).

### 3.5 Blood biochemical parameters

**Figure 1:**
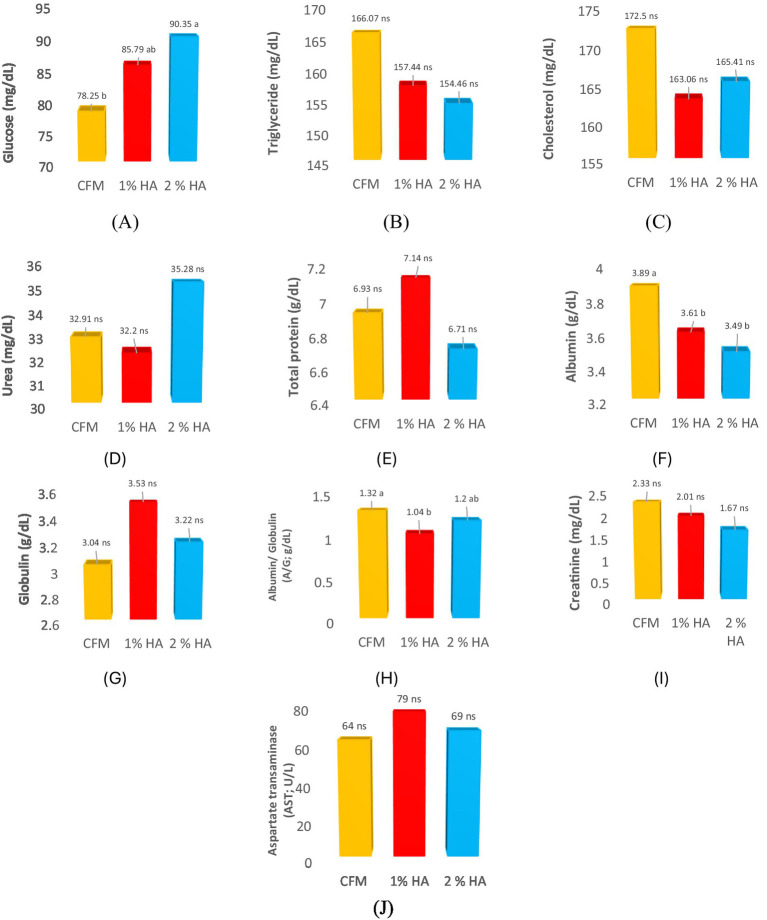
Selected blood biochemical parameters in the calves supplemented with 1 and 2% humic acid compared to the CFM group. **(A)** Glucose (mg/dL), **(B)** Triglyceride (mg/dL), **(C)** Cholesterol (mg/dL), **(D)** Urea (mg/dL), **(E)** Total protein (g/dL), **(F)** Albumin (g/dL), **(G)** Globulin (g/dL), **(H)** Albumin/Globulin (A/G; g/dL), **(I)** Creatinine (mg/dL), and **(J)** Aspartate transaminase (AST; U/L).

The results presented in Figure 1 show that a higher (*p* = 0.049) glucose concentration (mg/dL) was observed in the 2% HA group than in the CFM group, while the 1% HA group was not significantly different from the CFM group (Figure 1A). Furthermore, the HA-supplemented groups had lower (*p* = 0.002) albumin (g/dL) concentrations compared to the CFM group (Figure 1F). In addition, the albumin/globulin (A/G; g/dL) ratio was lower (*p* = 0.041) in the 1% HA group than in the CFM group (Figure 1H). No significant difference (*p* > 0.05) was observed in triglycerides (mg/dL), cholesterol (mg/dL), urea (mg/dL), total protein (g/dL), globulin (g/dL), creatinine (mg/dL), or aspartate transaminase (AST; U/L) among the groups (Figures 1B–D,G).

## 4 Discussion

The present study demonstrated a positive response to humic acid (HA) supplementation in concentrate feeds. It also tested the hypothesis that adding 1 and 2% HA improves overall growth performance and feed efficiency in calves by enhancing nutrient digestibility, optimizing ruminal fermentation patterns, and stimulating ruminal enzyme activity and certain blood biochemical parameters. Humic substances have been reported to enhance digestive efficiency by stabilizing rumen microflora, increasing enzyme activity, and promoting nutrient absorption (40). Dry matter intake (DMI) was lower in the 1% HA and 2% HA groups compared to the CFM group. The FCR based on dry matter, TDN, and DCP was improved in the treatment groups compared to the control group. This finding is consistent with Dorantes-Iturbide (41), who emphasized that the palatability of feed additives can lead to reduced intake due to strong odors or tastes, causing temporary feed refusal or sorting behavior. Animals tend to adjust their forage and concentrate intake to achieve energy balance. However, the absence of statistically significant changes in the digestibility coefficients of other nutrients, such as dry matter (DM), organic matter (OM), and crude fiber (CF), may reflect the dose-dependent or context-dependent effects of HA. Furthermore, El-Zaiat et al. (42) reported similar results in Barki goats, where supplementation with 2 g/day of HA did not significantly alter the apparent digestibility of major nutrients. Moreover, HA had no significant impact on *in vitro* nutrient digestibility, despite improving ruminal fermentation characteristics (19). The study found no significant differences in pH values among groups. These findings agree with those of Malyugina and Horky (43). However, HA may enhance ruminal environmental stability and generate ideal circumstances for microbial fermentation (19). The results showed significantly higher digestibility of crude protein (DCP) and ether extract (EE) in the 1% HA and 2% HA groups compared to the CFM group. Furthermore, HA promotes the growth of fibrolytic bacteria by stabilizing pH (44). These bacteria generate acetate and butyrate by fermenting structural carbohydrates, leading to a positive effect of HA characterized by a higher acetate-to-propionate ratio or increased production of acetate and butyrate. The results revealed that ammonia nitrogen (NH₃-N) was significantly lower in the 1% HA and 2% HA groups compared to the CFM group. Humic substances, with their ammonia-binding properties, can enhance microbial nitrogen utilization by promoting the growth of rumen microorganisms that incorporate ammonia into microbial protein (42). The results showed a significant improvement in the volatile fatty acid profile and a reduction in the rumen ammonia concentration, along with a significant stimulation of the activity of a group of digestive enzymes. According to El-Zaiat et al. (42), HA may enhance microbial protein synthesis and enzyme secretion by creating a more favorable environment for the rumen and stimulating rumen microbes. Increased activity of ruminal enzymes (*α*-amylase, cellulase, lipase, and protease) is a direct indicator of microbial protein synthesis. This can improve nitrogen efficiency and reduce environmental nitrogen losses, potentially enhancing animal performance (44). However, this contradicts previous studies showing increased NH₃-N concentrations at specific HA inclusion rates (43). The study found significant differences in the molar proportions of various VFAs among the groups, with the 2% HA group having higher acetate and propionate proportions and lower n-butyrate and iso-butyrate proportions compared to the 1% HA and CFM groups. HA does not directly stimulate ruminal fermentation in calves, but it may modulate specific microbial fermentation pathways, with increased acetate and propionate production indicating improved ruminal fermentation efficiency and greater energy availability for the host animal (45). However, no significant difference was found in the molar proportions of other VFAs. Elevated iso-butyrate concentrations in the HA-supplemented groups suggest increased deamination of branched-chain amino acids, while the lack of change in iso-valerate and valerate levels suggests that HA selectively influences certain fermentation networks (46). In addition, HA improves ruminal fermentation by shifting the microbial population toward a more efficient profile, resulting in a change in the molar ratios of volatile fatty acids (19). The protozoal community, influenced by HA, may be targeted to modulate genera such as *Entodinium*, which are known for their roles in bacterial predation and nitrogen recycling (47). This targeted shift could partially explain the reduction in ruminal ammonia nitrogen concentrations, while other genera, such as *Epidinium*, may decline due to HA’s mild antimicrobial activity (46). The increased activities of *α*-amylase, lipase, urease, and protease in the calves receiving HA supplementation indicate that HA may enhance enzyme activity in the rumen by influencing microbial populations. This observation aligns with the findings of El-Zaiat et al. (42), who suggested that HA creates a more suitable environment for beneficial microbes to thrive and produce digestive enzymes. Furthermore, cellulase activity was significantly higher in the 1% HA group compared to the CFM group. These findings are consistent with those of Sallam et al. (46) and Malyugina (43). The simultaneous increase in glucose and decrease in albumin likely reflects an improved metabolic state in HA-treated calves. Improved ruminal fermentation led to an increased energy supply (48), while protein resources were actively directed toward tissue building rather than being stored in the blood, resulting in a slight decrease in serum albumin (48).

## 5 Conclusion

In conclusion, these findings support the use of 1 and 2% HA supplements to improve nutrient digestibility and ruminal fermentation in fattening calves. HA enhanced feed conversion efficiency. Dietary supplementation with 1% or 2% humic acid improved ether extract digestibility by 15% and reduced ruminal ammonia-N by 40% while elevating acetate and propionate proportions compared to the control diet. These changes were accompanied by increased ruminal enzyme activity, indicating enhanced digestive capability. Blood biochemical profiles verified the safety of humic acid administration, with no negative health impacts observed. Overall, humic acid is recommended as a natural feed additive to improve digestibility and promote sustainable beef production.

## Data Availability

The original contributions presented in the study are included in the article/supplementary material, further inquiries can be directed to the corresponding author.
